# Antioxidant treatment enhances human mesenchymal stem cell anti-stress ability and therapeutic efficacy in an acute liver failure model

**DOI:** 10.1038/srep11100

**Published:** 2015-06-09

**Authors:** Wen Zeng, Jia Xiao, Gang Zheng, Feiyue Xing, George L. Tipoe, Xiaogang Wang, Chengyi He, Zhi-Ying Chen, Yingxia Liu

**Affiliations:** 1 State key Discipline of Infectious Diseases, Shenzhen Third People’s Hospital, Shenzhen, China; 2 Laboratory for Gene and Cell Therapy, Shenzhen Institute of Advanced Technology, Chinese Academy of Sciences, Shenzhen, China; 3 Department of Immunobiology, Institute of Tissue Transplantation and Immunology, Jinan University, Guangzhou, China; 4 Department of Anatomy, The University of Hong Kong, Hong Kong, China

## Abstract

One of the major problems influencing the therapeutic efficacy of stem cell therapy is the poor cell survival following transplantation. This is partly attributed to insufficient resistance of transplanted stem cells to oxidative and inflammatory stresses at the injured sites. In the current study, we demonstrated the pivotal role of antioxidant levels in human umbilical cord mesenchymal stem cells (hUCMSCs) dynamic *in vitro* anti-stress abilities against lipopolysaccharide (LPS)/H_2_O_2_ intoxication and *in vivo* therapeutic efficacy in a murine acute liver failure model induced by D-galactosamine/LPS (Gal/LPS) by either reducing the antioxidant levels with diethyl maleate (DEM) or increasing antioxidant levels with edaravone. Both the anti- and pro-oxidant treatments dramatically influenced the survival, apoptosis, and reactive oxygen species (ROS) production of hUCMSCs through the MAPK-PKC-Nrf2 pathway *in vitro*. When compared with untreated and DEM-treated cells, edaravone-treated hUCMSCs rescued NOD/SCID mice from Gal/LPS-induced death, significantly improved hepatic functions and promoted host liver regeneration. These effects were probably from increased stem cell homing, promoted proliferation, decreased apoptosis and enhanced secretion of hepatocyte growth factor (HGF) under hepatic stress environment. In conclusion, elevating levels of antioxidants in hUCMSCs with edaravone can significantly influence their hepatic tissue repair capacity.

In clinic, for the therapy of acute and chronic end-stage liver diseases, including acute liver failure, cirrhosis and liver cancer, transplantation is the gold standard procedure. However, severe global shortage of donor organs and possible immunological rejection significantly restrict its wide application^1^. Among various alternative therapeutic strategies, replacing damaged host hepatocytes and stimulating endogenous liver regenerative processes with transplanted stem cells hold promising prospect^2^,^3^. Currently, successful disease improvements by stem cell therapy have been achieved in drug-induced liver injury^4^, acute liver failure^5^, alcoholic liver disorders^6^, non-alcoholic fatty liver disease^7^, and liver fibrosis^8^. For the treatment of liver cancer, there are only a limited number of trials that used stem cells to treat hepatocellular carcinoma (HCC) and the results are quite controversial^9^.

Although stem cell therapy in the liver exhibits a variety of advantages such as pluripotency, self-renewal, ease of harvest, and minimal immunogenicity, in both pre-clinical studies and clinical trials, poor cell survival following transplantation continues to limit the efficacy of this therapy^10^,^11^. This is predominantly attributed to the inflammatory and oxidative stress environment at the site of injury^12^. It has been proposed that endogenous antioxidant level of stem cells could influence their fate after transplantation at injured host sites. For example, endothelial progenitor cells are shown to express high level of antioxidant enzymes and to have increased abilities of DNA repair as compared to more differentiated endothelial cells. Therefore, they are less sensitive to oxidative stress–induced apoptosis^13^. Similarly, after treatment with antioxidant N-acetylcysteine, muscle-derived stem cells (MDSCs) showed increased survival ratio and better ameliorative effects on an acute murine model of myocardial infarction^14^. However, information regarding the dynamic effects and mechanisms of stem cell endogenous antioxidant level on their anti-stress ability against exogenous stresses, particularly on liver disease is lacking. Moreover, the *in vitro* treatment agent, dose, and time-window are extremely important determinants influencing *in vivo* transplantation. In this study, we tested treatment time-windows of antioxidant edaravone and pro-oxidant diethyl maleate (DEM) in human umbilical cord mesenchymal stem cells (hUCMSCs) against lipopolysaccharide (LPS)/H_2_O_2_ challenge. The ameliorative effects and mechanisms of edaravone- or DEM-treated hUCMSCs on a murine acute liver failure model were then examined.

## Methods

### Reagents and antibodies

Edaravone (3-methyl-1-phenyl-2-pyrazolin-5-one) was purchased from Mitsubishi Pharma Corporation (Tokyo, Japan). DEM, D-galactosamine (Gal), LPS, and methylthiazolyldiphenyl-tetrazolium bromide (MTT) were products from Sigma-Aldrich (St Louis, MO). PD98059 and staurosporine were products of Calbiochem (Billerica, MA). All cell culture consumables and reagents were bought from either Corning Incorporated (Corning, NY) or Gibco (Carlsbad, CA). Antibodies against catalase (CAT), SOD1, phosphorylated p38 MAPK at Thr180/Tyr182, total p38 MAPK, phosphorylated ERK at Thr202/Tyr204, total ERK, and β-actin were bought from Cell Signaling (Beverly, MA).

### Isolation and expansion of hUCMSCs

Procedures for hUCMSCs isolation and expansion were performed as previously described^15^. All clinical procedures followed the protocols approved by the ethical committee of Shenzhen Institute of Advanced Technology, Chinese Academy of Sciences. All participants provided their written consents for the current study.

### Cellular treatments

hUCMSCs from passages 2 were seeded in 24-well plates with confluence around 60%–70% before treatment in a regulator cell CO_2_ incubator. Cells were divided into 5 groups (n = 4): (1) control group: no additional treatment; (2) oxidative/inflammatory stress (LPS) group: cells were treated with 0.1 μg/ml LPS and 200 μM H_2_O_2_ simultaneously; (3) LPS+Eda10 group: 2 hours before LPS/H_2_O_2_ treatment, cells were pre-incubated with 10 μM edaravone; (4) LPS+Eda20 group: 2 hours before LPS/H_2_O_2_ treatment, cells were pre-incubated with 20 μM edaravone; (5) LPS+DEM group: 2 hours before LPS/H_2_O_2_ treatment, cells were pre-incubated with 50 μM DEM. To delineate the optimal treating combination of dosage and duration, we collected treated cells at 12, 24, 36, 48, 60, and 72 hours post-treatment. It should be noted that 200 μM H_2_O_2_ is a relatively high concentration compared to levels observed *in vivo* during inflammation (~5 μM). We used this concentration in the current study to compensate for the lack of other reactive oxygen species (ROS) and pro-inflammatory cytokines observed in injured host tissues^14^.

### MTT assay

Cell viability was evaluated by the conversion of MTT to a purple color product by cellular mitochondria. After drug treatment, cells from each group were washed by sterile PBS 3 times and then incubated with 5 mg/ml MTT for 3 hours, and subsequently dissolved in dimethyl sulfoxide (DMSO). The absorbance of MTT was measured at 570 nm.

### Apoptotic ratio measurements

After drug treatment, Hoechst 33342 (5 μg/ml) and propidium iodide (5 μg/ml) were added to each well to stain live cells. The results were expressed as the percentage of apoptosis (PA): PA = apoptotic cell number/ total cell number × 100%^16^.

### Caspase-3/7 activity measurements

Activities of caspases-3/7 from cell lysates after treatments were measured using Cell Meter Caspase 3/7 Activity Apoptosis Assay Kit (AAT Bio., Sunnyvale, CA) according to the user manual. Final results were read at 520 nm in a micro-plate reader (Bio-Rad) and expressed as fold change in caspase 3/7 activity from control.

### ROS staining

Intracellular production of ROS was detected by fluorescence probe 2’,7’-dichloro-fluorescin diacetate (DCFH-DA, Sigma-Aldrich) as previously described^17^. Briefly, after treatment, cells were washed three times with PBS and then incubated in 10 μM DCFH-DA for 30 min at 37 °C for green fluorescent light visualization. Quantification of green fluorescence was analyzed by using ImageJ (Version 1.48, National Institutes of Health, Bethesda, MD).

### RNA extraction and quantitative PCR assay

Total RNA of cells was extracted by using illustra^TM^ RNAspin mini kit (GE healthcare, UK). The preparation of the first-strand cDNA was conducted following the instruction of the SuperScript^TM^ First-Strand Synthesis System (Invitrogen, Calsbad, CA). The mRNA expression levels of Bcl-2, Bax1, NAD(P)H:quinone oxidoreductase-1 (NQO-1), malic enzyme-1 (ME-1), oncostatin M (OSM) and epidermal growth factor (EGF) (for sequence information, see Supplementary Table 1) were measured by Takara SYBR premix Taq quantitative PCR system (Takara Bio Inc, Shiga, Japan) and in MyiQ2 real-time PCR machine (Bio-Rad, Hercules, CA). Parallel amplification of glyceraldehyde-3-phosphate dehydrogenase (GAPDH) was used as the internal control. Relative quantification was done by using the 2^−ΔΔCt^ method. The relative expression of the specific gene to the internal control was obtained and then expressed as percentage of the control value. All real-time PCR procedures including the design of primers, validation of PCR environment and quantification methods were performed according the MIQE guideline^18^.

### Cellular protein extraction, Western blotting, and Nrf2 activity assay

At each treatment time-point, cells were washed with sterile PBS for 3 times and then subjected to cytosolic and nuclear protein extraction by using a NE-PER Nuclear and Cytoplasmic Extraction System (Pierce, Rockford, IL). Protein samples were then quantified with BCA method from Bio-Rad. Western blot analyses of cell lysates were performed as described^19^. Parallel blotting of β-actin was used as the internal control.

To further investigate the mechanism of endogenous antioxidant level change, the nuclear protein of each sample was subjected to the measurement of Nrf2 transcription factor activity assay by using a commercial kit from Cayman Chemical Company (Ann Arbor, MI).

### GSH/GSSG ratio measurements

To measure the intracellular oxidative status of stem cells, the ratio between reduced glutathione (GSH) to oxidized glutathione (GSSG) of each cellular protein sample was measured by using a GSH/GSSG detection assay kit from Abcam (Cambridge, England).

### Inhibition of the ERK, PKC, and Keap1 pathways

To further investigate the underlying mechanisms contributing to the endogenous antioxidant level of hUCMSCs after edaravone or DEM pre-treatment, we firstly transiently silenced the expression of Keap1, the repressor of Nrf2 activation using its specific siRNA combination (Santa Cruz BioTechnology, Santa Cruz, CA). Transfection of Keap1 siRNA (100 nM) was conducted 1-day before the pre-treatment with DEM by using Lipofectamine 3000 (Invitrogen, Carlsbad, CA). Given the function of the ERK and PKC pathway in oxidative stress progression, the role of ERK/MAPK or PKC signaling following edaravone treatment was evaluated. That is, 1-hour before the edaravone (20 μM) incubation, cells were treated with 25 μM PD98059 (specific ERK/MAPK inhibitor) or 10 nM staurosporine (specific PKC inhibitor). Then cells were subjected to LPS/H_2_O_2_ challenge as previously described.

### Animal experiments

All animal experiments, including procedures, sampling and animal cares, in the current study were approved by and in accordance with guidelines and regulations from the ethical committee of Shenzhen Institute of Advanced Technology, Chinese Academy of Sciences. Male 6-week old (~20 g) non-obese diabetic severe combined immune-deficient (NOD/SCID) mice were bought from Guangdong Experimental Animal Center (Guangzhou, China). Mice were randomly divided into 8 groups (n = 12): (1) control group: mice were intraperitoneally (i.p.) injected with PBS only; (2) Gal/LPS group: mice were i.p. injected with 600 mg/kg Gal and 8 μg/kg LPS dissolved in PBS simultaneously; (3–5) vehicle-stem cell groups: mice were injected through tail-vein (t.v.) with 2 × 106 hUCMSCs (untreated, 20 μM edaravone-pretreated, and 50 μM DEM-pretreated, respectively) at passage 2; (6–8) Gal/LPS-stem cell groups: mice received 600 mg/kg Gal and 8 μg/kg LPS via i.p. injection, followed 6-hour later by 2 × 10^6^ hUCMSCs (untreated, 20 μM edaravone-pretreated, and 50 μM DEM-pretreated, respectively) at passage 2 through t.v. injection. The dosage combination of Gal and LPS, as well as the delivery route of stem cells were selected based on our previous study^20^. Murine serum was collected at day 1, 3, and 7 post-transplantation. Liver samples were collected at the end of the 7-day experiment and stored at –80 °C until further processing.

### Serum and liver tissue analysis

Serum was collected by centrifugation from whole blood sample at 1,000 *x*g for 10 min at 4 °C and stored at –80 °C. Liver tissue samples were fixed in 10% phosphate-buffered formalin, processed for histology and embedded in paraffin blocks. Five-micrometer tissue sections were cut and stained with hematoxylin and eosin (H&E).

### Serum ALT and AST assay

To evaluate the hepatic injury at the enzymatic level, serum ALT and AST levels were measured by using ALT (SGPT) and AST (SGOT) reagent sets (Teco diagnostics, Anaheim, CA) according to manufacturer’s instructions.

### Genomic DNA extraction and quantitative real-time PCR

To quantify the transplanted hUCMSCs and i-Heps that homed at the mice liver, a recently established real-time PCR quantification system has been used in the current study^21^. Briefly, genomic DNA at day 7 post-treatment was extracted from mouse livers using QIAamp genomic DNA extraction kit (Qiagen, Hilden, Germany). A pair of primers (see Supplementary Table 1 for sequence information) that generate a 141-bp fragment of human Down syndrome region at chromosome 21 were used to quantify the human-derived cells. The real-time PCR reaction was performed using an ABI 7500 real-time PCR system (Applied Biosystems, Foster City, CA) for 40 cycles with denaturing at 95 °C for 30 seconds and annealing at 63 °C for 34 seconds, with a SYBR-Green Realtime PCR mix (Takara, Dalian, China).

### PKH labeling and fluorescent microscopy

The fluorescent dye PKH26 has been used as the cell tracer to locate the transplanted stem cells in host animal^22^. Before transplantation, hUCMSCs at passage 2 were labeled with the PKH26 MINI kit (Sigma-Aldrich) according to the manufacture’s suggestions and previously reported protocol^23^. When animals were sacrificed, liver tissues were cryopreserved in optimal cutting temperature medium (OCT, TissueTek, SakuraAmericas, Torrence, CA). Frozen tissue sections (5 μm) were collected on glass slides and fixed with 100% methanol for 10 min at 4 °C, then washed in PBS for 10 min at room temperature. Local hepatic cells were mounted using DAPI mounting solution (Beyotime Biotechnology, Jiangsu, China) and used to normalize the expansion percentage of transplanted stem cells.

### hUCMSCs proliferation and apoptosis following transplantation

After 7-day post-transplantation to the injured NOD/SCID mice liver, donor hUCMSCs proliferation was quantified by immunohistochemical staining of PCNA. Fresh liver tissues were embedded with OCT medium and “snap-frozen” in dry ice. Frozen sections of 10-μm thickness were prepared and subjected to permeabilization in acetone at –20 °C for 10 min. To reduce non-specific signal, slides were incubated with goat serum blocking buffer (Boster, Wuhan, China) at room temperature for 1-hour. Subsequently, the slides were incubated with primary antibodies PCNA (1:100, Cell Signaling). After washing thrice with PBS, slides were incubated with mouse antibody against mouse IgG conjugated with Alexa flour (1:1000, Cell Signaling). Sections were co-stained with human cytokeratin-18 (hCK-18; 1:100, Abcam HK, NT, HK) and goat antibody against rabbit IgG conjugated with FITC (1:1000, Abcam HK). Apoptosis was quantified by terminal dUPT nick end-labeling (TUNEL) using ApopTag Plus Peroxidase *In Situ* Apoptosis Detection Kit (Chemicon, Billerica, MA) after 3-day post-transplantation. The number of PCNA cells or apoptotic cells was quantified in 3 microscopic fields at ×40 magnification using ImageJ software.

### Serum ELISA assay

Serum level of TNF-α and IL-6 from each mouse was measured by using ELISA kits from PeproTech (Rocky Hill, NJ) according to the manufacturer’s instructions.

### *In vitro* and *in vivo* secretion of hepatocyte growth factor (HGF) by hUCMSCs

For the evaluation of HGF secretion by hUCMSCs *in vitro*, cells at passage 3 were received pre-treatment with edaravone or DEM as described above and then washed thrice with Ca2^+^ and Mg2^+^-free PBS (Sigma-Aldrich), and cultured in 10 ml DMEM supplemented with 0.05% bovine serum albumin (BSA, Sigma-Aldrich) for 24 hours. After that, collected and concentrated conditioned medium was subjected to HGF ELISA measurement (RayBioteck, Norcross, GA). For *in vivo* measurements, paraffin embedded liver tissues at day 7 post-challenge were prepared for immunohistochemical staining of HGF. HGF-secreting cells were labeled with HRP/DAB system (Zhongqiao, Beijing, China). Hematoxylin was used as the counterstain of cellular nuclei.

### Statistical analysis

Data from each group were expressed as means ± SEM. Statistical comparison between groups was done using the Kruskal–Wallis test followed by Dunn’s post hoc test to detect differences in all groups. A value of p < 0.05 was considered to be statistically significant (Prism 5.0, Graphpad software, Inc., San Diego, CA).

## Results

### Edaravone improved hUCMSCs viability and morphology after oxidative/inflammatory challenge

**Figure 1:**
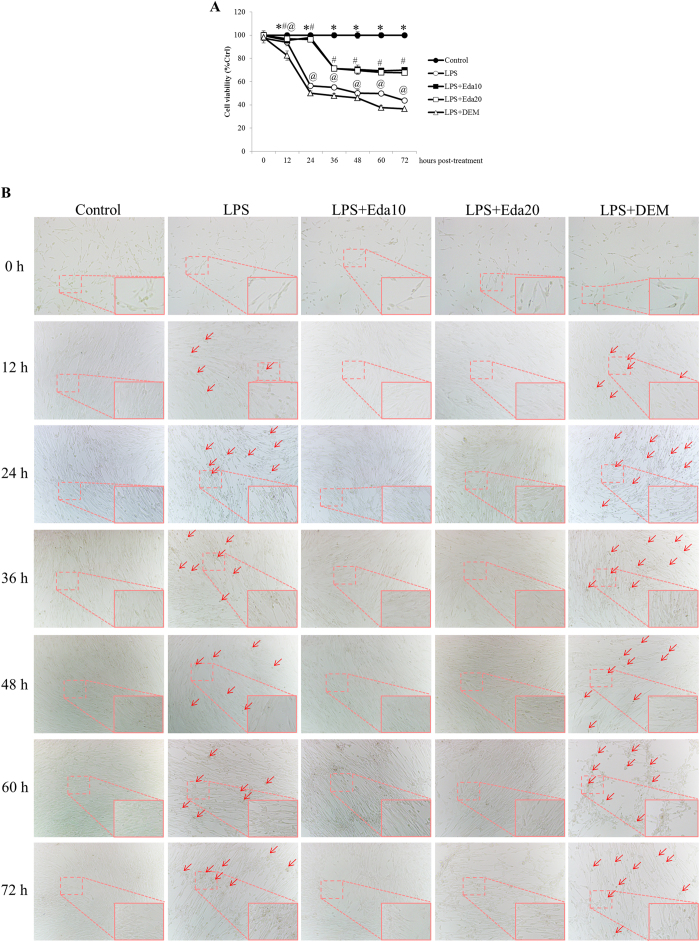
*In vitro* dynamic effects of edaravone and diethyl maleate (DEM) treatments of human umbilical cord mesenchymal stem cells (hUCMSCs) on cell viability and morphology after lipopolysaccharide (LPS)/H_2_O_2_ intoxication (n = 4). (**a**) Edaravone treatment significantly increased the cell viability damaged by LPS/H_2_O_2_ challenge. DEM treatment exacerbated the cell viability. “*” means significant changes (*P* < 0.05) between control and treatments; “#” means significant changes (*P* < 0.05) between edaravone treatment groups (10 μM and 20 μM) and LPS/H_2_O_2_ group; “^@^” means significant change (*P* < 0.05) between DEM-treated group and LPS/H_2_O_2_ group. (**b**) Edaravone improved hUCMSCs cell morphology damaged by LPS/H_2_O_2_ challenge while DEM further exacerbated it. Arrows indicate typical abnormal hUCMSCs. LPS, LPS/H_2_O_2_ challenge; Eda10, 10 μM edaravone; Eda20, 20 μM edaravone. Magnification 200x (Magnified part 400x).

Similar to the *in vivo* situation, potent oxidative/inflammatory stress *in vitro* (LPS/H_2_O_2_ incubation) caused evident decrease of cell viability, particularly after 24 hours of the challenge (*P* < 0.05; Fig. 1A). Pre-treatment with pro-oxidant DEM further reduced the viability of stem cells challenged by LPS/H_2_O_2_ at all time-points post-treatment (*P* < 0.05). As expected, both concentration of edaravone significantly recovered the cell viability impaired by the challenge of LPS/H_2_O_2_ (*P* < 0.05; Fig. 1A). There was no significant change of cell viability between 10 μM and 20 μM edaravone pre-treated hUCMSCs (*P* > 0.05). In consistent with the viability results, LPS/H_2_O_2_ challenge resulted in obvious morphological change of hUCMSCs, including cell shrinkage and occurrence of apoptosis (Fig. 1B). Pre-treatment with DEM significantly increased the number of abnormal cells, while edaravone recovered the cell morphology (Fig. 1B).

### Increased endogenous antioxidant level attenuated stem cell apoptosis

**Figure 2:**
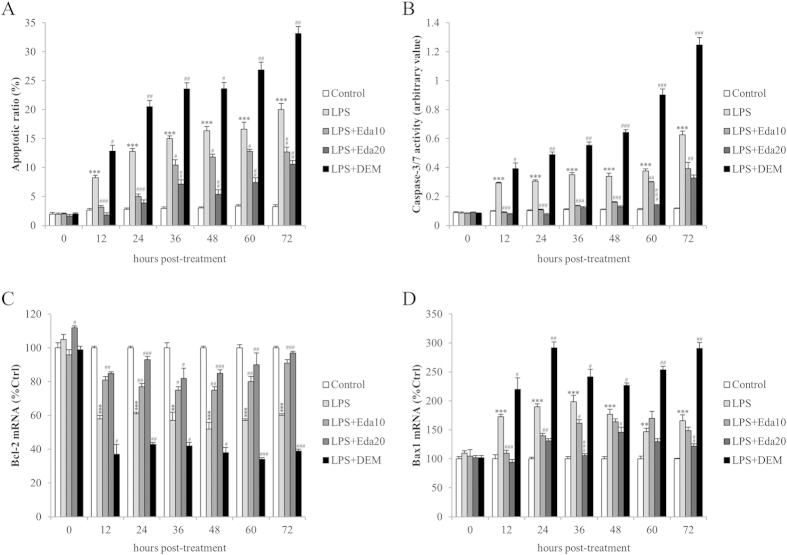
*In vitro* dynamic effects of edaravone and diethyl maleate (DEM) treatments of human umbilical cord mesenchymal stem cells (hUCMSCs) on cell apoptosis after lipopolysaccharide (LPS)/H_2_O_2_ intoxication (n = 4). (**a**) Apoptotic ratio, (**b**) caspase-3/7 activity, (**c**) cellular Bcl-2 mRNA expression and (**d**) cellular Bax1 mRNA expression of each group of hUCMSCs after different durations of LPS/H_2_O_2_ challenge. LPS, LPS/H_2_O_2_ challenge; Eda10, 10 μM edaravone; Eda20, 20 μM edaravone. “**” means *P* < 0.01 against control group; “***” means *P* < 0.001 against control group; “#” means *P* < 0.05 against LPS/H_2_O_2_ group; “##” means *P* < 0.01 against LPS/H_2_O_2_ group; “###” means *P* < 0.001 against LPS/H_2_O_2_ group.

To investigate the effects and mechanisms of endogenous antioxidant level on the pathogenesis of apoptosis under oxidative/inflammatory stress, the apoptotic ratio and corresponding caspase-3/7 activity were examined at each time point after LPS/H_2_O_2_ challenge. It was shown that the basal apoptotic ratio slightly increased from the beginning of the experiment to 72-hour post-treatment (~2.0% vs. ~3.6%, *P* < 0.05). LPS/H_2_O_2_ challenge significantly increased the cellular apoptosis as time prolonging (*P* < 0.001). Pre-treatment with edaravone (10 μM and 20 μM) or DEM significantly decreased or further increased the apoptosis of hUCMSCs, respectively (*P* < 0.05 or <0.001; Fig. 2A). The activity change of cellular caspase-3/7 was consistent with the change of cellular apoptotic ratio (Fig. 2B). To confirm the involvement of intrinsic apoptotic pathway in this study, the mRNA expressional changes of Bcl-2 and Bax1 were then quantified by quantitative PCR. Results exhibited that, when compared with the control group, LPS/H_2_O_2_ challenge significantly down-regulated the mRNA level of anti-apoptotic molecule Bcl-2, while up-regulated the mRNA level of pro-apoptotic molecule Bax1 at 12, 24, 36, 48, 60, 72-hour post-treatment. Pre-treatment with both concentrations of edaravone abolished such effects. DEM pre-incubation further reduced the level of Bcl-2 and increased the level of Bax1 after LPS/H_2_O_2_ challenge, indicating an exacerbated status of stem cell apoptosis (Fig. 2C,D).

### Edaravone attenuated cellular ROS production after oxidative/inflammatory challenge

**Figure 3:**
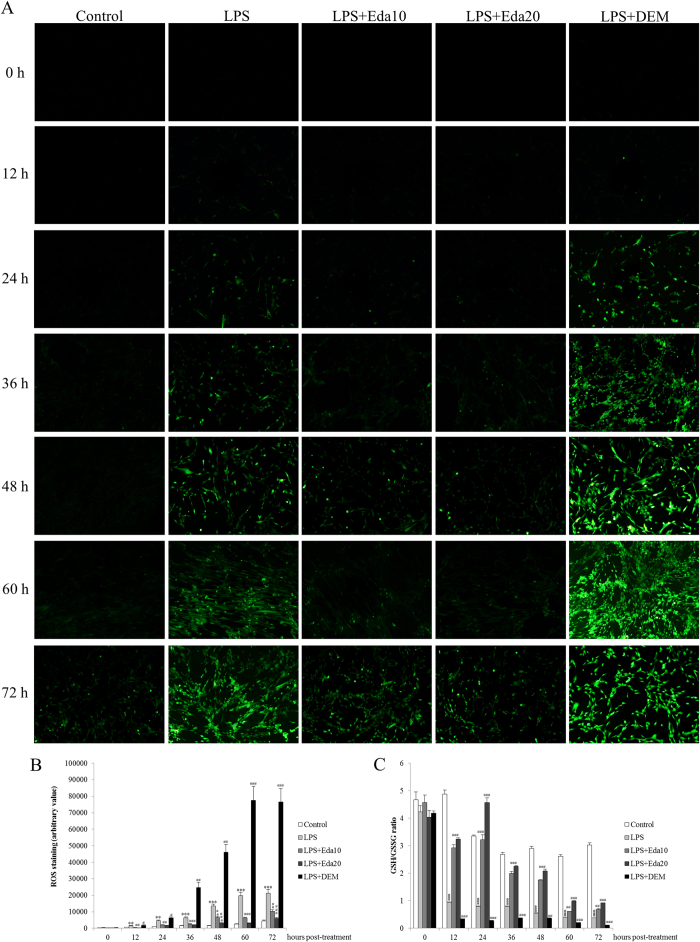
*In vitro* dynamic effects of edaravone and diethyl maleate (DEM) treatments of human umbilical cord mesenchymal stem cells (hUCMSCs) on reactive oxygen species (ROS) production and GSH/GSSG ratio change after lipopolysaccharide (LPS)/H_2_O_2_ intoxication (n = 4). (**a**) The production of ROS was stained by DCFH-DA of each group of hUCMSCs after different durations of LPS/H_2_O_2_ challenge. (**b**) Quantification of ROS production. (**c**) Cellular GSH/GSSG change of each group of hUCMSCs was examined by commercial kit. LPS, LPS/H_2_O_2_ challenge; Eda10, 10 μM edaravone; Eda20, 20 μM edaravone. Magnification 200x. “**” means *P* < 0.01 against control group; “***” means *P* < 0.001 against control group; “#” means *P* < 0.05 against LPS/H_2_O_2_ group; “##” means *P* < 0.01 against LPS/H_2_O_2_ group; “###” means *P* < 0.001 against LPS/H_2_O_2_ group.

To directly demonstrate the antioxidant effects of edaravone on LPS/H_2_O_2_-caused oxidative stress, DCFH-DA staining of hUCMSCs was applied to show the change of cellular ROS production. From 24-hour post-treatment afterwards, LPS/H_2_O_2_-induced obvious DCFH-DA positive signals in the culture medium, which was attenuated by edaravone pre-treatment but worsened by DEM pre-treatment (Fig. 3A,B). In cells, a decreased ratio of GSH/GSSG is an indication of oxidative stress, since when cells are exposed to increased levels of oxidative stress, oxidized glutathione (GSSG) accumulates and the reduced form (GSH) decreases^24^. In line with the ROS production, LPS/H_2_O_2_ challenge caused significant decrease of cellular GSH/GSSG ratio when compared with that of the control group at 12, 24, 36, 48, 60, and 72 hours post-treatment. Such decreases were evidently restored by edaravone (10 μM and 20 μM) but further reduced by DEM (Fig. 3C). It should be noted that the restoration of GSH/GSSG by edaravone is incomplete and appeared to be deteriorated at longer time points of 60 and 72 h, probably due to the decreased and desensitized endogenous antioxidant pathway of stem cells in response to exogenous antioxidant stimulation, which needs further investigation.

### Edaravone restored levels of endogenous antioxidant enzymes impaired by oxidative/inflammatory challenge

**Figure 4:**
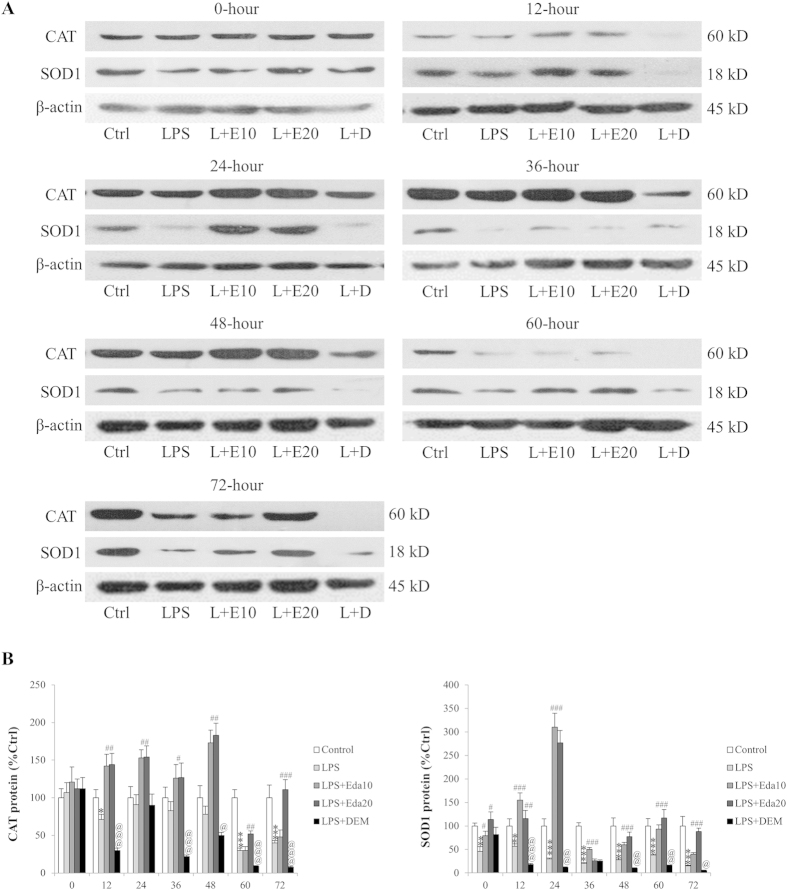
*In vitr*o dynamic effects of edaravone and diethyl maleate (DEM) treatments of human umbilical cord mesenchymal stem cells (hUCMSCs) on antioxidant enzymes (catalase, CAT and superoxide dismutase-1, SOD1) expression after lipopolysaccharide (LPS)/H_2_O_2_ intoxication (n = 4). The protein expression of both CAT and SOD1 was measured by Western blot (**a**) and normalized by internal control β-actin (**b**). Quantified data were conducted by using ImageJ. LPS, LPS/H_2_O_2_ challenge; L+E10, LPS/H_2_O_2_ + 10 μM edaravone; L+E20, LPS/H_2_O_2_ + 20 μM edaravone; L+D, LPS/H_2_O_2_ + DEM. “*” “**” “***” mean significant changes (*P* < 0.05, 0.01, 0.001) between control and treatments, respectively; “#” “##” “###” mean significant changes (*P* < 0.05, 0.01, 0.001) between edaravone treatment group (10 μM or 20 μM) and LPS/H_2_O_2_ group, respectively; “@” “@@” “@@@” mean significant change (*P* < 0.05, 0.01, 0.001) between DEM-treated group and LPS/H_2_O_2_ group, respectively.

CAT and SOD1 are important endogenous antioxidant enzymes against intracellular and extracellular oxidative stress^25^. Western blotting results suggested that at 12, 24, 36, 48, 60, and 72 hours after LPS/H_2_O_2_ treatment, the protein expression levels of both CAT and SOD1 were significantly down-regulated (particularly for SOD1; Fig. 4). Edaravone pre-treatment significantly restored their expression levels while DEM pre-incubation reduced them to lower levels (*P* < 0.05; Fig. 4), indicating that the redox regulating effects of edaravone and DEM were directly associated with the modulation of endogenous antioxidant enzymes.

### Edaravone and DEM influence stem cell antioxidant level through regulating MAPK, PKC and Nrf2 pathways

**Figure 5:**
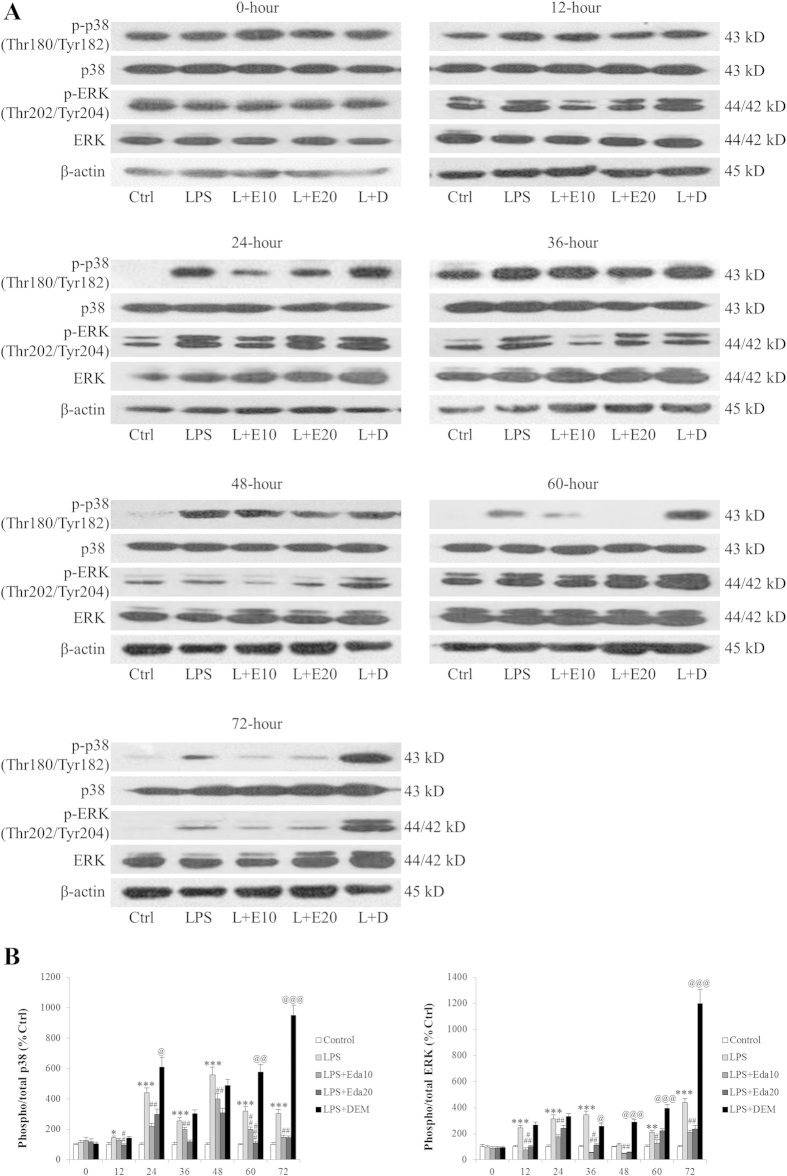
*In vitro* dynamic effects of edaravone and diethyl maleate (DEM) treatments of human umbilical cord mesenchymal stem cells (hUCMSCs) on phosphorylated and total MAPKs (p38 MAPK and ERK1/2) expression after lipopolysaccharide (LPS)/H_2_O_2_ intoxication (n = 4). The protein expression of both p38 MAPK and ERK1/2 was measured by Western blot (**a**) and normalized by internal control β-actin (**b**). Quantified data were conducted by using ImageJ. LPS, LPS/H_2_O_2_ challenge; L+E10, LPS/H_2_O_2_ + 10 μM edaravone; L+E20, LPS/H_2_O_2_ + 20 μM edaravone; L+D, LPS/H_2_O_2_ + DEM. “*” “**” “***” mean significant changes (*P* < 0.05, 0.01, 0.001) between control and treatments, respectively; “##” “###” mean significant changes (*P* < 0.01, 0.001) between edaravone treatment group (10 μM or 20 μM) and LPS/H_2_O_2_ group, respectively; “@” “@@” “@@@” mean significant change (*P* < 0.05, 0.01, 0.001) between DEM-treated group and LPS/H_2_O_2_ group, respectively.

**Figure 6:**
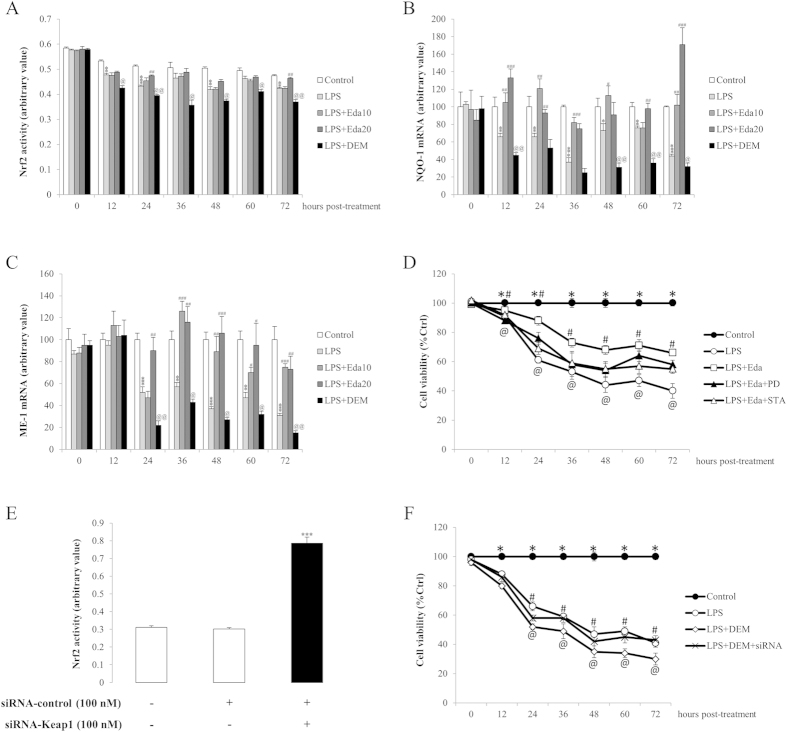
*In vitro* dynamic effects of edaravone and diethyl maleate (DEM) treatments of human umbilical cord mesenchymal stem cells (hUCMSCs) on Nrf2 expression and its upstream/downstream pathway after lipopolysaccharide (LPS)/H_2_O_2_ intoxication (n = 4). (**a**) The activity change of Nrf2 of hUCMSCs was measured by a commercial kit from nuclear protein. (**b**,**c**) The mRNA expressional change of NAD(P)H:quinone oxidoreductase-1 (NQO-1) and malic enzyme-1 (ME-1) measured by quantitative PCR. (**d**) After the application MAPK inhibitor PD98059 (25 μM) and PKC inhibitor staurosporine (10 nM), the beneficial effects of 20 μM edaravone on cell viability were partially abolished. (**e**) Silence of Keap1 by specific siRNA significantly increased the Nrf2 activity. (**f**) Enhancement of Nrf2 activity by Keap1 silence partially reversed the detrimental effects of DEM on cell viability. “*” “**” “***” mean significant changes (*P* < 0.05, 0.01, 0.001) between control and treatments, respectively; “#” “##” “###” mean significant changes (*P* < 0.05, 0.01, 0.001) between edaravone treatment group (10 μM or 20 μM) and LPS/H_2_O_2_ group, respectively; “@” “@@” mean significant change (*P* < 0.05, 0.01) between DEM-treated group and LPS/H_2_O_2_ group, respectively. LPS, LPS/H_2_O_2_ challenge; Eda10, 10 μM edaravone; Eda20, 20 μM edaravone; PD, PD98059; STA, staurosporine.

Under oxidative stress, MAPK and PKC pathways are activated to degrade Keap1, the repressor of transcription factor Nrf2, leading to the activation of Nrf2 and downstream antioxidant processes in the cell^26^. To test the involvement of these mechanisms in the antioxidant status change of hUCMSCs, we firstly measured the change of the MAPK pathway. Results showed that LPS/H_2_O_2_ treatment potentiated the phosphorylation of both p38 MAPK and ERK1/2 at most time points post-treatment without influencing their total protein expression (Fig. 5). In agreement with previous results, pre-treatment with edaravone or DEM significantly abolished or further strengthened the effects of LPS/H_2_O_2_ treatment, respectively (Fig. 5). Then the transcriptional activity change of Nrf2 was assessed. It was found that at 12, 24, 36, 48, 60, and 72 hours after LPS/H_2_O_2_ treatment, the activity of Nrf2 was significantly decreased when compared with the control group (Fig. 6A). Application of 20 μM, but not 10 μM edaravone partially restored the activity. Pre-incubation with DEM, as expected, further reduced the activity of Nrf2 (Fig. 6A). In addition, the mRNA expression of Nrf2 downstream antioxidant genes, NQO-1 and ME-1 was also down-regulated by LPS/H_2_O_2_ challenge at most of the treatment time points, which was in agreement with the results of CAT/SOD1 protein expression. Edaravone and DEM further differentiately regulated their expressions (Fig. 6B,C). Under oxidative/inflammatory stress conditions (LPS/H_2_O_2_ exposure), the inhibition of the ERK or PKC pathway, using PD98059 or staurosporine, respectively, caused a significant decrease in the cell survival of edaravone-treated hUCMSCs compared to uninhibited edaravone-treated hUCMSCs (Fig. 6D). Vehicle-PD98059 or staurosporine treatment in hUCMSCs only slightly reduced their survival rates when compared to rates of uninhibited, hUCMSCs, indicating that the decrease of cell viability was not attributed to the a direct toxicity of these agents (data not shown). Then we examined the effects of Keap1 silence (which causes the enhancement of Nrf2 activity) on DEM-treated hUCMSCs. Twenty four hours after the Keap1 siRNA transfection, the activity of Nrf2 in hUCMSCs was significantly higher than that of un-transfected or transfected with control siRNA cells (*P* < 0.001; Fig. 6E). After the LPS/H_2_O_2_ exposure, the inhibition of Keap1 partially restored the cell viability that impaired by the pre-incubation with DEM (Fig. 6F), indicating an essential involvement of Nrf2 in the antioxidant properties of hUCMSCs.

### *In vivo* hUCMSCs engraftment and survival in an acute liver injury model

**Figure 7:**
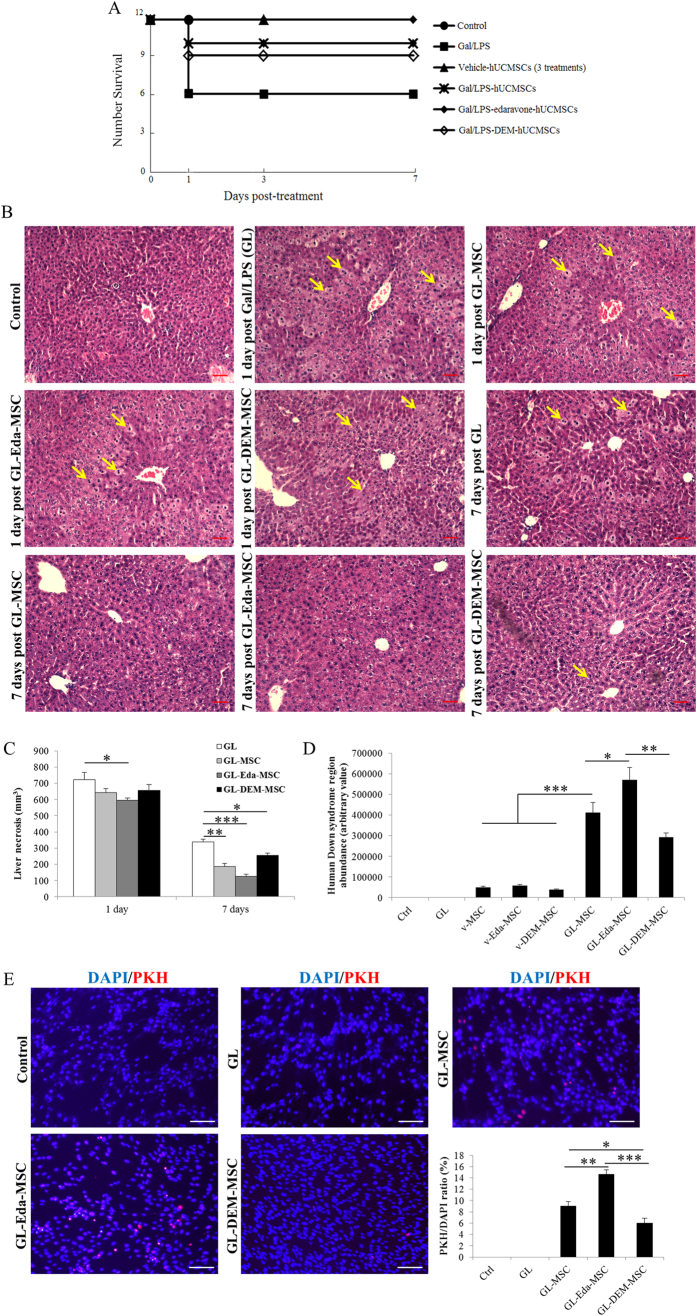
*In vivo* effects of edaravone and diethyl maleate (DEM) treatments of human umbilical cord mesenchymal stem cells (hUCMSCs) on their therapeutic efficacy in an acute liver failure model induced by D-galactosamine/LPS (Gal/LPS). (**a**) Edaravone treatment showed better rescuing effects on NOD/SCID mice death than untreated and DEM-treated hUCMSCs. (**b**,**c**) Edaravone treatment showed better ameliorative effects on hepatic histology than untreated and DEM-treated hUCMSCs after liver failure. Yellow arrows indicate typical necro-inflammatory cells. (**d**) Edaravone increased while DEM decreased the expanded cell number in the host liver after transplantation. (**e**) Localization of hUCMSCs in the Gal/LPS-challenged mouse liver using PKH immunofluorescnece at day 7 post-infusion. Quantified data also proved the finding. GL, Gal/LPS. Magnification 200x. Bar: 20 μm. “*” “**” “***” mean significant changes (*P* < 0.05, 0.01, 0.001) between indicated two groups, respectively.

Seven days after the Gal/LPS intoxication of NOD/SCID mice, 50% of mice tested (n = 12) survived. For those mice with co-injection of hUCMSCs, only 2 mice were dead. Edaravone-pretreatment successfully rescued all mice. In the group of DEM-pretreated prior to Gal/LPS intoxication, however, 3 mice died during the experiment (Fig. 7A). Gal/LPS treatment is known to induce hepatocyte necrosis and inflammatory responses. In the current study, Gal/LPS treatment alone caused evident hepatic necrosis in the NOD/SCID mice in 24-hour (Fig. 7B). Administration of edaravone-pretreated stem cells partially alleviated such hepatic injury (Fig. 7B,C). Seven days after the treatment, edaravone-pretreated stem cells showed the best ameliorative effects while DEM-pretreated stem cells exhibited only minimal therapeutic effects, when compared to the non-stem cell injected Gal/LPS group (Fig. 7B,C). In the vehicle control groups, injection of either hUCMSCs or edaravone/DEM pre-treated hUCMSCs showed no significant changes on the liver morphology throughout the experiment (data not shown).

To quantify the human-derived cells engrafted in the NOD/SCID mice liver, the ratio between human gene (Down Syndrome Region Sequence) and host genome at the end of the experiment (7 days) was determined by using quantitative real-time PCR. It was found that vehicle stem cell groups (no pre-treatment, edaravone or DEM pre-treated) only generated a small amount of human gene (Fig. 7D). Transplantation with edaravone-pretreated hUCMSCs following the Gal/LPS intoxication showed the highest abundance of human gene, while DEM-pretreated hUCMSCs showed significantly less human gene than that of non-treated hUCMSCs after Gal/LPS intoxication (Fig. 7D). These results suggested that administration of hUCMSCs after acute liver injury could accelerate the host hepatic regenerative process. Edaravone pre-treatment significantly improved the therapeutic effects and engraftment efficacy of stem cells while DEM pre-treatment impaired these effects. In addition, recruitment of hUCMSCs into the mice liver was also examined by PKH immunofluorescence (IF) and further proved that edaravone improved the expansion efficiency of stem cells after acute liver failure (Fig. 7E).

### Transplantation of hUCMSCs improved serum biochemistry

**Figure 8:**
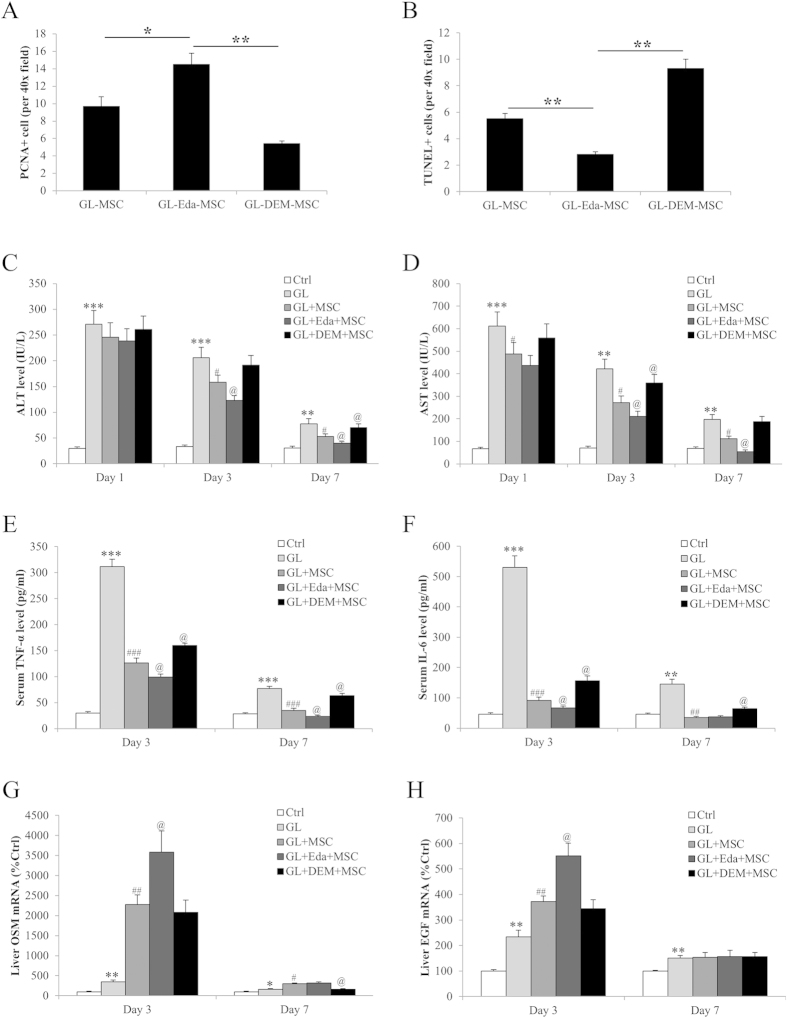
*In vivo* effects of edaravone and diethyl maleate (DEM) treatments of human umbilical cord mesenchymal stem cells (hUCMSCs) on serum markers and liver regeneration in an acute liver failure model induced by D-galactosamine/LPS (Gal/LPS). (**a**,**b**) Edaravone treatment promoted proliferation and decreased apoptosis of transplanted hUCMSCs in the murine liver. (**c**,**d**) Edaravone further improved the ameliorative effects of hUCMSCs on serum aminotransferases level. (**e**,**f**) Edaravone further reduced the production of pro-inflammatory cytokines after liver failure. (**g**,**h**) Edaravone further promoted the host liver regeneration after liver failure. “*” “**” “***” mean significant changes (*P* < 0.05, 0.01, 0.001) between control and treatments, respectively; “#” “##” “###” mean significant changes (*P* < 0.05, 0.01, 0.001) between edaravone treatment group (10 μM or 20 μM) and LPS/H_2_O_2_ group, respectively; “@” means significant change (*P* < 0.05) between DEM-treated group and LPS/H_2_O_2_ group, respectively. GL, Gal/LPS.

To examine the influence of antioxidant status in stem cell proliferation and apoptosis after transplantation, we quantified the number of PCNA+ cells or apoptotic cells (with co-staining of hCK-18) per ×40 high-powered field. It was shown that, when compared with untreated hUCMSCs, pre-treatment with edaravone significantly increased the PCNA+ cell number but reduced the apoptotic cell number, indicating a potentiated resistance of transplanted hUCMSCs against host liver stresses (Fig. 8A,B).

To further study the influence of edaravone or DEM on the therapeutic effects of hUCMSCs on Gal/LPS induced acute liver injury, changes of serum ALT and AST levels in each group of mice were evaluated at 1-, 3-, and 7-day post-injection of Gal/LPS challenge. One-day and 3-day after the Gal/LPS challenge, both ALT and AST levels in the serum of all Gal/LPS-treated mice increased significantly as compared to those of the untreated control mice. However, transplantation of hUCMSCs significantly reduced the serum concentrations of these liver injury indicators (Fig. 8C,D). In consistent with the histological results, edaravone pre-treated hUCMSCs showed stronger reducing ability of both ALT and AST while DEM pre-treatment significantly impaired the alleviation (Fig. 8C,D). Then the serum levels of pro-inflammatory cytokines, TNF-α and IL-6, were examined by ELISA assay in each group of mice at day 3 and day 7 post-treatment. Transplantation with hUCMSCs significantly reduced the pro-inflammatory secretion induced by Gal/LPS treatment at both time points, indicating an attenuated status of body inflammation. Edaravone further improved the amelioration of inflammation while DEM treatment exacerbated it (Fig. 8E,F). At last, we measured the hepatic expressional changes of liver regeneration-related genes, OSM and EGF mice at day 3 and day 7 post-treatment. At day 3, treatment with Gal/LPS significantly induced the expression of these two genes, which was further enhanced by the transplantation with hUCMSCs. As expected, edaravone further enhanced the expression while DEM reduced it (Fig. 8G,H). At day 7, the expressional change of these genes was not as obvious as day 3.

### Infused hUCMSCs ameliorated host hepatic injury partly through secreting HGF

**Figure 9:**
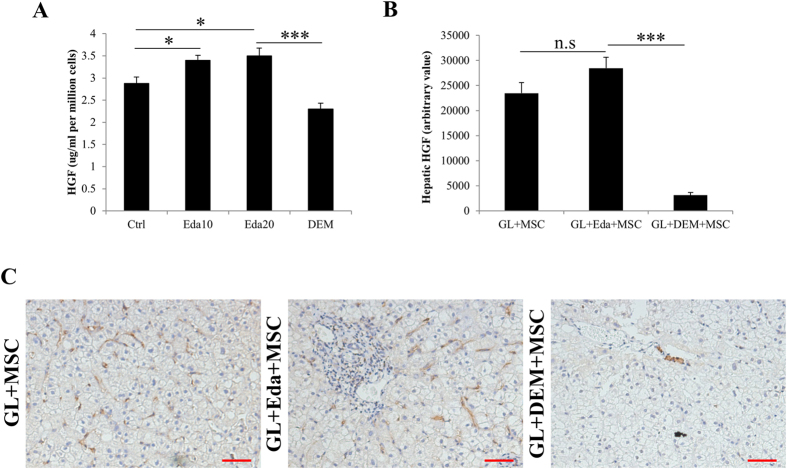
Assessment of secretion and expression of human hepatocyte growth factor (HGF) by human umbilical cord mesenchymal stem cells (hUCMSCs) *in vitro* and *in vivo*. (**a**) *In vitro* measurements of HGF secretion by hUCMSCs with or without pre-treatment by edaravone or diethyl maleate (DEM). (**b**,**c**) Representative *in vivo* image of HGF-positive cells by infused hUCMSCs in the host mouse liver at day 7 post challenge. Eda10, 10 μM edaravone; Eda20, 20 μM edaravone; GL, D-galactosamine/LPS. “*” “***” mean significant changes (*P* < 0.05, 0.001) between indicated two groups, respectively. Bar: 50 μm.

To elucidate the possible mechanisms of the ameliorated role of transplanted stem cells in the host liver, we firstly evaluated the secretion level change of HGF by cultured hUCMSCs. It was shown that naïve hUCMSCs secreted measurable level of HGF to the culture medium. Pre-treatment with edaravone slightly promoted such secretion while DEM showed opposite effects (Fig. 9A). Furthermore, we found that after transplantation, infused stem cells were capable of secreting HGF in the host liver. DEM treatment impaired such paracrine actions (Fig. 9B,C).

## Discussion

Stem cell-based therapy has been recognized as a promising treating strategy of a variety of diseases, including liver disorders. For example, bone marrow MSCs provide protection against liver injury by antioxidative process, vasculature protection, hepatocyte differentiation, and trophic effects^27^. However, the efficacy of these therapies is below expectations^28^. One of the main reasons for this is the low survival ratio of transplanted stem cells at injured sites because of harsh oxidative stress and inflammatory environment^29^. To counter these effects, a number of pre-treatment methods began to emerge. For example, it was reported that pre-treatment with antioxidant N-acetylcysteine (NAC) significantly improved cell survival ratio of muscle-derived stem cells (MDSCs) and cardiac function in an acute murine model of myocardial infarction^14^. A recent study also found that treatment with melatonin, a common antioxidant, further improved adipose-derived mesenchymal stem cell (ADSC) therapy for acute interstitial cystitis in rat^30^. Thus, enhancement of endogenous antioxidant level of stem cells before transplantation seems to be a strategy to improve the therapeutic efficacy.

We have previously identified that hUCMSCs, which are easily accessible and multipotent, exhibited evident repairing effects on an acute liver failure murine model induced by Gal/LPS^15^. Although these hUCMSCs and trans-differentiated i-Heps rescued mice with improved hepatic functions, the repair process remains limited. To increase the repair efficacy, in the current study, we firstly used edaravone as antioxidant and DEM, a nontoxic chemical that binds to GSH and inactivates it, to reduce antioxidant levels^31^, as pro-oxidant to examine the dynamic changes of cellular endogenous antioxidant level, viability, and apoptosis. From 24-hour post-treatment, hUCMSCs started to show significant loss of viability, with increased ROS production and cellular apoptosis. Edaravone significantly counteracted such effects while DEM further exacerbated them, including the regulation of antioxidant enzymes and apoptotic genes. This result is consistent with other studies using NAC or melatonin^14^,^30^. It was extensively studied that MAPK and PKC pathways could directly modulate the transcriptional activity of Nrf2, which plays antioxidant roles by controlling the transcription of downstream genes^32^,^33^. We also found that the beneficial effects of edaravone on hUCMSCs were through decrease of phosphorylated p38 MAPK and ERK1/2, as well as increase of Nrf2 activity and its downstream antioxidant enzyme expression (Figs 5 and 6). Application of MAPK inhibitor PD98059 or PKC inhibitor staurosporine reversed the improvement by edaravone. Silence of Nrf2 inhibitory protein – Keap1 partially abolished the detrimental effects of DEM. These results confirmed that the modulation of stem cell endogenous antioxidant level was, at least partly, through the MAPK-PKC-Nrf2 pathway. This is confirmed by a very recent study showing that dynamic changes in intracellular ROS levels regulate stem cell homeostasis through Nrf2-dependent signaling^34^. It should be noted that in the current study, expression of antioxidant enzymes (CAT, SOD1, NQO-1 and ME-1) and Nrf2 activity were down-regulated by oxidant treatment, which was in line with several recent studies in stem cells and hepatocytes^35^,^36^,^37^. Some other reports, however, found that antioxidant genes are increased to survive when cells are exposed to oxidative stress inducers^30^,^38^,^39^. This difference might be attributed to the defensive nature of human MSCs to oxidative stress through expressing high basal level of active forms of CAT, glutathione peroxidase (GPx), and SOD, which confers the resistance against acute ROS-mediated cellular damage^40^. Addition of toxin or oxidant may impair this defense wall to maintain the relatively low level of antioxidant enzymes and Nrf2 activity, resulting in the oxidative stress status of the stem cells.

In line with the *in vitro* findings, when compared with untreated hUCMSCs, edaravone pre-treated stem cells exhibited improved therapeutic properties, including enhanced survival ratio of the mice, improved hepatic histology, reduced serum aminotransferases/cytokines and promoted liver regeneration (Figs 7 and 8). This can be from increased transplanted stem cell number, which is probably from induced cell proliferation and inhibited apoptosis by the pre-treatment with edaravone. In contrast, DEM-treated hUCMSCs showed minimal therapeutic effects on acute liver failure. These results strongly suggested that the antioxidant status of hUCMSCs before transplantation is vital for the functional tissue repair of acute liver failure^14^.

Indeed, there are several limitations of the current study. Firstly, we only investigated the involvement of MAPK, PKC and Nrf2 in the regulation of stem cell antioxidant status. Functions of other oxidative stress-related pathways, such as PI3K/Akt and FoxO/TXNIP need further studies. Secondly, mechanisms that influence the enhanced repairing efficacy of stem cell after transplantation are only partly examined. We proved that increased number of transplanted hUCMSCs secreted more HGF to promote the recovery of adjacent host liver cells and this action was also influenced by the antioxidant and pro-oxidant treatments (Fig. 9), which was consistent with previous reports^41^,^42^. Thirdly, regardless of the fact that MSCs are proven with minimal immunogenicity and low tumorigenesis^43^, the safety of drug-treated stem cell transplantation needs long-term observation in animal models and clinical trials. In a long-term approach in NOD/SCID mice, the safe and efficient use of MSCs by injection not revealed side effect^44^.

ROS are increasingly recognized as important signaling molecules involved in gene regulation of stem cells^45^. Since oxidative stress, inflammation, and necro-apoptosis are typical consequences of acute liver failure which holds high possibility to cause death^46^, transplantation with enhanced anti-oxidative ability stem cells may significantly improve the therapeutic efficacy in clinical trials. Thus, reducing the *in vitro* culture duration^15^ and maintaining relatively high antioxidant endogenous level of isolated stem cells (e.g. by clinically proven drug edaravone) before transplantation are possible strategies for future regenerative medicine applications. Furthermore, this study (1) provided useful information of the dynamic cellular changes at different time points after *in vitro* oxidative/inflammatory stress induction, which will assist future stem cell handling in both basic study and clinical trials; and (2) implied a new potent and widely used clinical drug, edaravone, as an useful antioxidant treating agent in stem cell preparation before transplantation.

## Additional Information

**How to cite this article**: Zeng, W. *et al.* Antioxidant treatment enhances human mesenchymal stem cell anti-stress ability and therapeutic efficacy in an acute liver failure model. *Sci. Rep.* **5**, 11100; doi: 10.1038/srep11100 (2015).

## Supplementary Material

Supplementary Information
