# Supplementary material for: Antioxidant treatment enhances human mesenchymal stem cell anti-stress ability and therapeutic efficacy in an acute liver failure model
Source: Sci Rep. 2015 Jun 9;5:11100. doi: 10.1038/srep11100 (PMC4460871; doi:10.1038/srep11100)

**Antioxidant treatment enhances human mesenchymal stem cell anti-stress ability and therapeutic efficacy in an acute liver failure model**

Wen Zeng^1,2*^, Jia Xiao^1,3,4*^, Gang Zheng^2^, Feiyue Xing^3^, George L. Tipoe^4^, Xiaogang Wang^3^, Chengyi He^2^, Zhi-Ying Chen^2^, & Yingxia Liu^1^

^1^State key Discipline of Infectious Diseases, Shenzhen Third People’s Hospital, Shenzhen, China; ^2^Laboratory for Gene and Cell Therapy, Shenzhen Institute of Advanced Technology, Chinese Academy of Sciences, Shenzhen, China; ^3^Department of Immunobiology, Institute of Tissue Transplantation and Immunology, Jinan University, Guangzhou, China; ^4^Department of Anatomy, The University of Hong Kong, Hong Kong, China

Supplementary Table 1: Sequence information of quantitative PCR analysis

| Name | Sequence (5’-3’) |
| --- | --- |
| Human Bcl-2 | Forward: GGTGGGGTCATGTGTGTGG |
|  | Reverse: CGGTTCAGGTACTCAGTCATCC |
| Human Bax1 | Forward: CCCGAGAGGTCTTTTTCCGAG |
|  | Reverse: CCAGCCCATGATGGTTCTGAT |
| Human NQO-1 | Forward: CCTCTATGCCATGAACTT |
|  | Reverse: TATAAGCCAGAACAGACTC |
| Human ME1 | Forward: CGGCAGAGAAGAGTAAGA |
|  | Reverse: ACTTGTTCAGGAGACGAA |
| Human Down syndrome region | Forward: ATGCTGATGTCTGGGTAGGGTG |
|  | Reverse: TGAGTCAGGAGCCAGCGTATG |
| Mouse OSM | Forward: CAGAATCAGGCGAACCTCACG |
|  | Reverse: AGCTCTCAGGTCAGGTGTGTT |
| Mouse EGF | Forward: TTAACGGGACAGGACTAGAGAAA |
|  | Reverse: AAGGAACTTAGAAGAACTCGGGA |
| Human GAPDH | Forward: CTGGGCTACACTGAGCACC |
|  | Reverse: AAGTGGTCGTTGAGGGCAATG |
| Mouse GAPDH | Forward: AGGTCGGTGTGAACGGATTTG |
|  | Reverse: TGTAGACCATGTAGTTGAGGTCA |


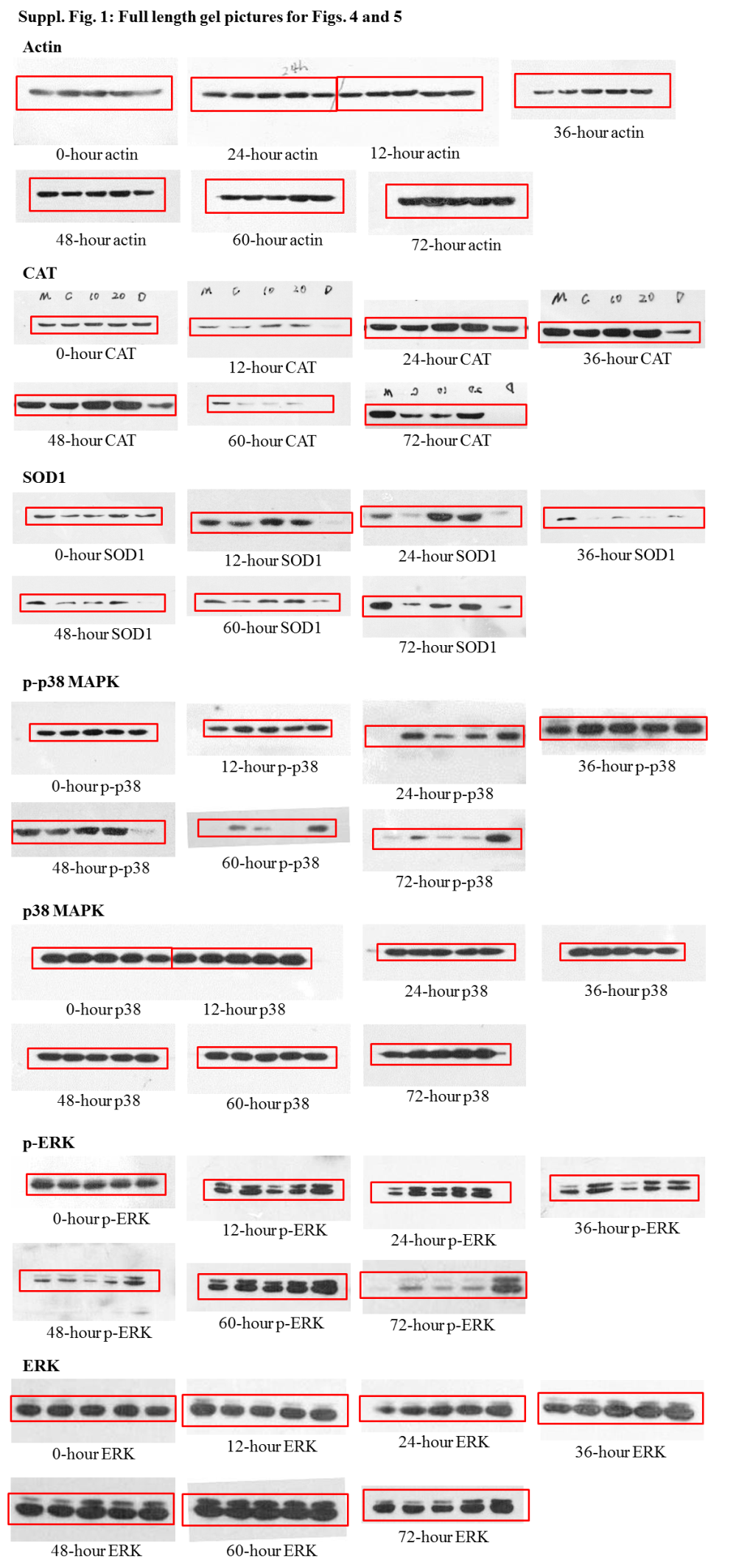

Supplement: Supplementary Information [file srep11100-s1.doc]
